# CT Characteristics of the Thymus in Adult Dogs with Non-Thymic Neoplasia Compared to Young Dogs

**DOI:** 10.3390/vetsci10030192

**Published:** 2023-03-04

**Authors:** Alessia Cordella, Jimmy H. Saunders, Emmelie Stock

**Affiliations:** Department of Morphology, Imaging, Orthopedics, Rehabilitation and Nutrition, Gent University, Salisburylaan 133, 9820 Merelbeke, Belgium

**Keywords:** thymus, thymic hyperplasia, computed tomography, thorax

## Abstract

**Simple Summary:**

The thymus is a lymphatic organ located in the cranial mediastinum. Both in humans and dogs, the thymus is largely changing with age, becoming smaller with time and also undergoing replacement of the active tissue with fat. Computed tomography is the imaging modality of election for the visualization and characterization of the thymus in human patients, and the characteristics of this organ with regard to the age of the patients are well described. On the contrary, in dogs, detailed description of the computed tomographic appearance of the thymus in adult and young patients is so far lacking. The results of this study show the different appearance of the thymus in two populations: adult dogs with diagnosed neoplasia and young dogs.

**Abstract:**

The thymus is a lymphatic mediastinal organ that is largely subject to changes with age. In human patients, the CT characteristics of the thymus in children and adults is well described. Furthermore, it is known in human medicine that stress can lead to a reduction in the size of the thymus, followed by a phase of hyperplasia (called the ‘rebound effect’). The visualization of thymic tissue in the cranial mediastinum of adult dogs with neoplasia is possible and could be related to a similar effect. In this study, we aimed to describe the CT characteristics of the thymus in adult dogs with neoplasia and to compare the aspect of the thymus in these dogs to juvenile dogs with a presumed normal thymus. A total of 11 adult dogs with neoplasia and 20 juvenile dogs were included. Several CT features of the thymus were evaluated, including the size, shape, and pre- and post-contrast attenuation values. The overall appearance was lobulated in all of the adult dogs and homogeneous in all of the juvenile dogs; it was left-sided in all of the adult dogs, while it was located in the midline in a few of the juvenile dogs (right-sided only in one). The thymus was less attenuating in adult dogs, in some cases with negative minimum pre-contrast attenuation values. In some dogs with neoplasia, the thymus can be detected at CT examination despite their age.

## 1. Introduction

The thymus is mainly a lymphoid structure located in the cranial mediastinum, with a role in T-lymphocyte maturation [1,2]. The size of the thymus in dogs differs with age; it is relatively large at birth, and it continues to grow reaching its maximum size around 4–6 months of age [2]. Then, the thymus progressively involutes, with it being no longer visible at about 1 year of age in most cases, and the lymphoid tissue is progressively replaced by fatty tissue [1,2,3]. Its visualization with different imaging modalities is therefore conditioned by the age of the patient [3,4].

Computed tomography is widely used nowadays to assess mediastinal structures in dogs, and the CT characteristics of different types of mediastinal masses have been previously described [5,6,7]. 

In humans, the CT characteristics of a normal thymus have been described both in children and adult patients. A normal thymus in children appears as a variably attenuating cranial mediastinal structure, with smooth lateral contours, without a tendency to produce displacement or deformity of the surrounding structures [8]. Thymic CT attenuation correlates inversely with age, likely representing the increase in the amount of fat [9]. In addition, the volume of the thymus decreases progressively with age in humans, with no solid tissue component left in the majority of patients older than 50 years old, where the involuted thymus is difficult to differentiate from the mediastinal fat even in CT [10,11].

Thymic hyperplasia has been reported in humans after recovery from stress, associated with thyroid diseases, and after chemotherapy [12,13,14,15]. This phenomenon is known as ‘rebound thymic hyperplasia’ and the mechanism is thymic depletion due to high plasma glucocorticoid concentrations, followed by a rebound effect when cortisol levels drop [16,17]. Little information is currently available in the veterinary literature regarding the normal CT appearance of the thymus in young and adult dogs and about thymic hyperplasia in this species [16]. 

The authors incidentally noticed the presence of a well-visible thymus in the cranial mediastinum of dogs that had undergone CT examination. The aim of this retrospective study was therefore to describe the CT characteristics of the thymus in this population of adult dogs and to compare them with those of a population of young dogs. 

## 2. Materials and Methods

This single-center descriptive study consisted in the retrospective description of cases in which the thymus was noticed in adult dogs at the time of CT examination. Dogs who underwent CT examination at Ghent University in the period between August 2020 and August 2022, in which the thymus was visible, were considered for inclusion. Dogs were included if they had a thoracic/whole body CT scan consisting of at least one pre-contrast scan and one post-contrast scan available for review, and if they had the confirmation of primary disease (thoracic or other than thoracic). Data regarding the age, sex, breed, and body weight of these dogs were collected, together with the final diagnosis of the primary disease. Exclusion criteria were incomplete patient data, incomplete or suboptimal CT studies, and a lack of definitive diagnosis. Young dogs (less than 9 months old), who underwent CT examination of the thorax for different clinical purposes in the same period of time, were also included for comparison. 

All CT images were retrieved from the Picture Archiving and Communication System (PACS) and analyzed using freestanding workstations (OsiriX v5.8.5 64-bit, Geneva, Switzerland) by a third-year European College of Veterinary Imaging resident (A.C.). Different features of the thymus were subjectively evaluated: (1) the shape (triangular, elongated, bilobed, or flattened,); (2) the overall appearance (lobulated or homogeneous); (3) the lateralization in the mediastinum (left-sided, midline, or right-sided); the grade and type of enhancement (mild/moderate/severe and homogeneous/heterogeneous). Quantitative findings, such as the dimensions (length, width, and height) and the pre- and post-contrast mean, minimum, and maximum attenuation values (measured in Hounsfield Units—HU) of the thymus were also assessed. In particular, to assess the attenuation values, a round region of interest (ROI) was placed (approximately) in the center of the structure in the pre-contrast images and copied (to have the exact same location and dimensions of the ROI) in the post-contrast images. When more than one post-contrast phase were available, the delayed phase was used for the measurements. 

### CT Scanning Techniques

Computed tomographic data were obtained with a 320-row MDCT unit (Aquilion One, Toshiba Medical Systems, Otawara, Japan). The technical parameters for all of the patients were as follows: helical modality, 120 kVp, 200 mAs, image matrix 512 × 512, 0.5 mm slice thickness. All of the dogs (except two in the young dog’s group) were scanned in sternal recumbency on the CT table, with the head first, front limbs cranially extended, and hindlimbs caudally extended. For all studies, a pre-contrast series of the whole body was acquired, followed by one post-contrast series acquired from 1 to 3 min after injection of iodinated contrast agent (iohexol 370 mgI/mL, 2 mLI/kg dosage followed by a saline flush) via the cephalic vein with a dual-barrel injector system. Some studies consisted of more post-contrast phases (either dual-phase or ECG-gated cardiac protocol), but the scans evaluated for all dogs included were pre-contrast and delayed post-contrast phases. 

## 3. Results

### 3.1. Animals

In the period of inclusion, 73 dogs who has undergone a thoracic CT received (or presented with) a definitive diagnosis of neoplasia (based on cytology, histology, or post-mortem). Of these, the thymus was clearly visible in the cranial mediastinum in 11 dogs (15%). The median age of the 11 dogs included was 9 (2–12) years, and the median body weight was 22.7 (7.2–82.3) kg. The majority of the included dogs were males (8/11—73%), of which 4 were neutered, and the remaining 3/11 (27%) were females; all three were neutered. Eleven different breeds were represented (one dog each): a Cavalier King Charles Spaniel, a White Swiss Shepherd, a Staffordshire Bull Terrier, a Shih-Tzu, a Jack Russell Terrier, a Mastino Napoletano, a Pug, a Chow Chow, an American Staffordshire Terrier, a Border Collie, and a crossbreed dog. 

All of the dogs included in this group (adult dogs) were diagnosed with neoplasia, and in particular, one dog had a meningioma at the level of T5, one had an anal sac adenocarcinoma, one had a mast cell tumor in the neck, one had a mammary gland carcinoma, one had soft tissue sarcoma in the cervical region, one had a nasal carcinoma, one had muscular hemangiosarcoma, one had Leydig cell tumor of the testicle, one had rib chondrosarcoma, and two had synovial cell sarcomas (one in the stifle and one in the tarsus). 

In the same period, 20 young dogs (less than 9 months old) underwent CT examination of the thorax. The median age of this group of dogs was 4 (2–9) months, and the median body weight was 13.5 (1.5–47) kg. Eleven out of the twenty dogs were males (55%), and the remaining 9/20 (45%) were females, all intact. Different breeds were represented: two Border Collies, two Chihuahuas, two English Bulldogs, two Golden Retrievers, one Bullmastiff, one Australian Cattle Dog, one Coton de Tulear, one Great Dane, one Bernese Mountain Dog, one Flatcoated Retriever, one French Bulldog, one Labrador Retriever, one Malinois, one Newfoundlander, one Thai Ridgeback, and one Dachshund. Seven of these dogs underwent CT examination for cardiac congenital anomalies (six with pulmonic stenosis and one with a ventricular septal defect), four for thoracic disease (two with pneumonia, one with pyothorax, and one with megaesophagus), four for orthopedic or neurological disease (one with a spinal cyst, one with meningitis/arthritis, one with hip and elbow dysplasia, and one with a dermoid sinus), three for trauma, one for abdominal disease (enteritis), and one presented with situs inversus. 

### 3.2. CT Appearance of the Thymus in Adult and Juvenile Dogs

CT features of the thymus in the adult dogs and in the juvenile dogs groups are summarized in Table 1.

**Figure 1:**
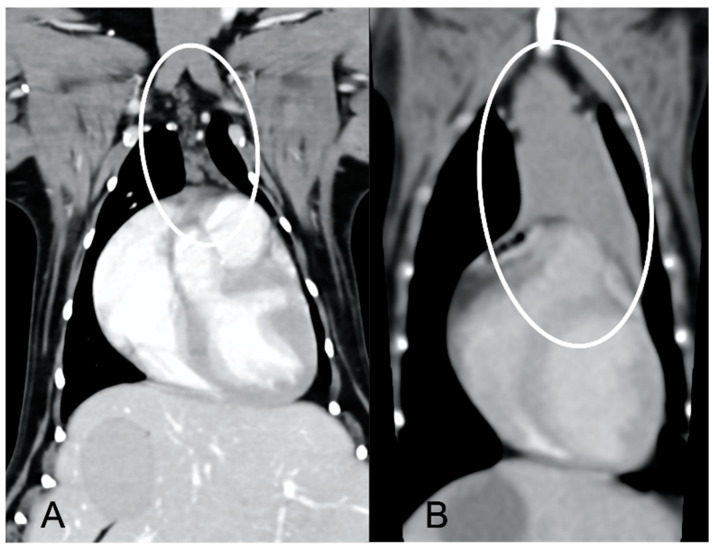
Shape of the thymus (circled) in an adult dog (**A**) compared to a young dog (**B**). Post-contrast dorsal CT reconstruction. Note the different relative sizes of the thymus in the two dogs, and the heterogeneous (lobulated) appearance of the thymus in (**A**) with respect to the thymus in (**B**). In both cases, the shape of the thymus is triangular.

**Figure 2:**
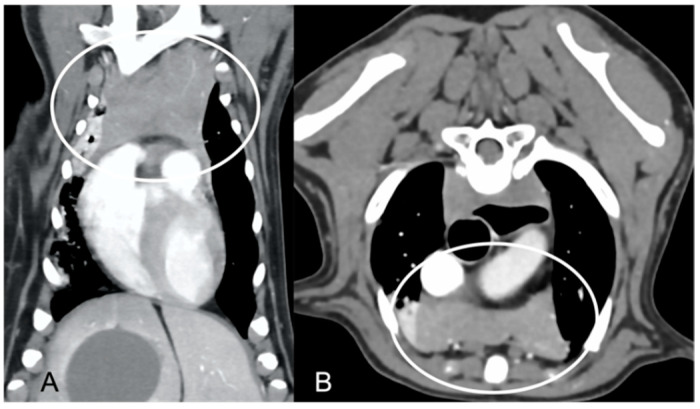
Example of a flattened thymus (circled) in a young dog. Post-contrast dorsal CT reconstruction (**A**) and transverse image (**B**) of the same patient. Note the flattened appearance of the thymus, enlarged in the laterolateral direction (width) and smaller in the dorsoventral direction (thickness).

In both groups, most of the dogs presented with a triangular thymus (Figure 1); in the adult group, some dogs had an elongated thymus, and in the juvenile group, some dogs had a flattened thymus (Figure 2).

**Figure 3:**
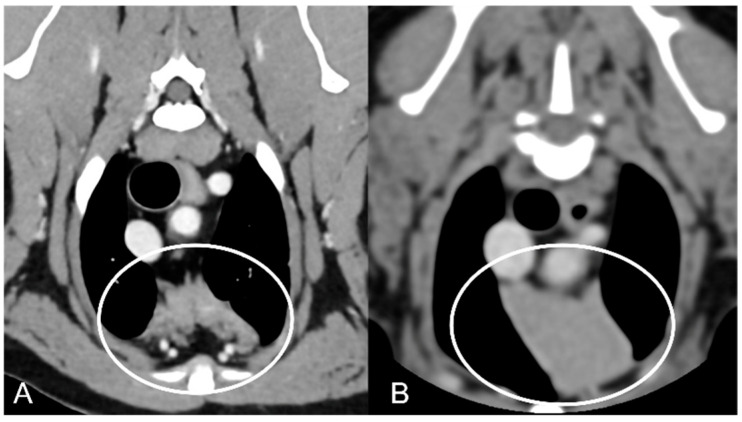
Appearance of the parenchyma of the thymus (circled) in an adult dog (**A**) compared to a young dog (**B**). Post-contrast transverse CT images. The appearance is lobulated in adult dogs (**A**) and homogeneous in young dogs (**B**).

**Figure 4:**
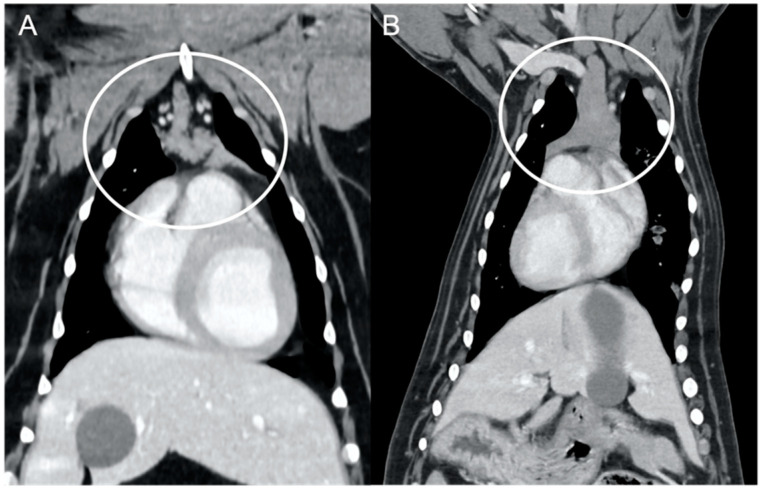
Lateralization of the thymus (circled) in two dogs. Post-contrast dorsal CT reconstructions. A mainly left-sided thymus in an adult dog (**A**) compared to a mainly right-sided thymus in a young dog (**B**) with situs inversus.

The overall appearance was lobulated in all of the adult dogs and homogeneous in all of the juvenile dogs (Figure 1 and Figure 3). The thymus was left-sided in all of the adult dogs, while it was located in the midline in a few juvenile dogs; one presented a thymus on the right side due to situs inversus (Figure 4). The maximum dimension of the thymus in the adult dogs group was always the length, while in some juvenile dogs, the maximum diameter was the width (Figure 2). The dimensions of the thymus were variable between different patients, with several breeds included and large differences in body weight between the dogs. For this reason, a ratio between the maximum diameter of the thymus (measured in cm) and the body weight (measured in kg) was calculated for each dog. The median ratio in the adult dogs group was 0.2 (minimum: 0.06; maximum: 0.4), while in juvenile dogs, it was 0.4 (minimum: 0.1; maximum: 1.4). The dogs with the higher ratio (>1) were in the juvenile group and they presented with spinal a cyst and enteritis and were 6 and 7 months old, respectively. The dogs with the lower ratio (<0.1) were in the adult group and they presented with muscular hemangiosarcoma (one dog) and synovial cell sarcoma (two dogs) and were 2, 6, and 8 years old, respectively. 

**Figure 5:**
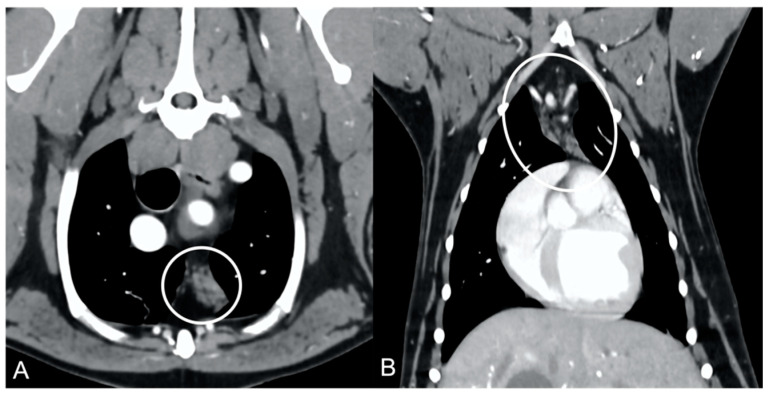
Appearance of the thymus (circled) in an adult dog. Post-contrast transverse CT image (**A**) and dorsal reconstruction (**B**). Note the presence of multiple hypoattenuating septi within the parenchyma, giving the thymus a lobulated appearance and overall low attenuation.

The thymus was less attenuating in adult dogs compared to young dogs, with a median of the mean pre- and post-contrast attenuation values lower in adult dogs compared to juvenile dogs (Table 1). In 5/11 (45%) adult dogs, the minimum pre-contrast attenuation values were negative values (from −22 to −2 HU), due to the presence of multiple, hypoattenuating, thick septi within the thymic parenchyma (Figure 5).

## 4. Discussion

The CT characteristics of the thymus in young and adult dogs have been described in this study. 

In young dogs, the thymus showed different shapes, with the majority presenting with a triangular appearance and some with flattened ones. In humans, a normal thymus can show some degree of individual variation, but some features are considered characteristic of a normal gland, including smooth lateral contours, convex lateral borders in very young patients, and straight or concave lateral borders with increasing age [8]. The sharp angular contour to the lateral margin of the thymus is similar to the described “sail sign” in thoracic radiography [8]. This radiographic feature has also been described in thoracic radiographs of young dogs [3,4], and the majority of young dogs in our population showed a similar shape in the dorsal reconstructions. The parenchyma of the thymus in our population of young dogs was homogeneous in all cases and with variable soft tissue attenuation values, which were higher in the post-contrast series. These characteristics were similar to those described in human medicine, where the thymus can show high variability in its attenuation in preadolescent and adolescent patients, especially in the pre-contrast series [8].

According to the results of this study, the thymus was less attenuating in adult dogs compared to young dogs, both in the pre- and post-contrast series. This finding is in accordance with the human literature, in which CT attenuation of the thymus is inversely correlated with age [9,10,17]. In particular, the decrease in attenuation is due to the known fatty degeneration that occurs in the thymus [8,9,17,18]. The increased amount of fat in the thymus of adult dogs compared to the younger population is also visible in our population, where almost half of the adult dogs show negative pre-contrast attenuation values, typical for fat tissue. In humans, other factors have been found to influence the attenuation of the thymus in adult patients, such as sex, cigarette smoking, and obesity [18,19]. The thymus in women shows less fat content than in men, most likely due to delayed thymic involution in women [18]. In our population there was a disproportion in the included adult dogs, with three-quarters of them being males; therefore, a comparison between sex was not attempted, and further studies are necessary to test this hypothesis in the canine species. Similarly, body condition score has not been evaluated as a factor in our study, but interestingly, upon retrospective revision, no patients with severely increased body condition scores were included in our population. 

The shape of the thymus in our population of adult dogs was variable, with the majority of the included dogs showing a triangular or elongated thymus, and only one with a bilobed thymus. In human patients, the thymus may appear as two separate lobes or as an arrowhead (triangle) formed by the confluence of the right and left lobes [17]. Furthermore, it has been suggested that in adult human patients (older than 40 years old), the presence of an ovoid or spherical soft tissue thymic appearance usually represents a neoplasm [10]. 

Several CT features of thymic neoplasia have been described in dogs. Thymomas have been described as large, space-occupying masses arising from the cranial mediastinum, frequently heterogeneous or with a cystic appearance, mainly left-sided but being more centrally located with increasing size [6]. Vascular invasions have been reported, especially in larger masses [6]. Cranial mediastinal lymphomas have been described as more homogeneous masses compared to thymic epithelial neoplasms, more likely to envelop the cranial vena cava [7]. The CT characteristics of thymic neoplasia reported in these previous studies, such as large masses, heterogeneous, enveloping or invading the adjacent vasculature [6,7], were significantly different from the findings of the current study, in which the thymus was in fact considered non-neoplastic. The CT appearance of thymic hyperplasia in humans can be variable, but some features, such as bipyramidal morphology and the presence of gross intercalated fat (also described as ‘marbling’), are considered pathognomonic [20]. This appearance of ‘marbling’, with the presence of several hypoattenuating septations throughout the thymic parenchyma, was present in all of the adult dogs included in the current study, suggesting that these dogs may have presented with thymic hyperplasia at the time of CT examination.

A histopathological, post-mortem study showed that thymic cysts are associated with several pathologic conditions in dogs, including neoplasia [21]. While the young and healthy dogs’ histology revealed a high proportion of lymphocytes, in adult chronically diseased cases, the cystic component was predominant [21]. The differences in histological components could reflect the different appearance of the thymus at CT examination in our two populations of dogs. The cystic component was not evident in CT in any of the cases included, but it is possible that some thymic cysts may have been missed, as it is known from human medicine that thymic cysts can have high attenuation values and resemble solid tissue on CT [20]. 

Despite the fact that a small thymic remnant can be detected in some dogs at any age [2], it has been reported that, in adult dogs, the thymus should have undergone the involution processes, and it is in most cases no longer visible at about 1 year of age [1,3]. The thymus was well visible in the adult dogs included in the current study. The characteristics, as already mentioned, were more suggestive of hyperplasia and not of a neoplastic process (neither a thymic epithelial tumor nor lymphoma). Rebound thymic hyperplasia has been rarely described in veterinary patients, with the CT features proposed being thymic enlargement but with a normal shape [18]. In humans, the thymus can atrophy during illness, and excessive regrowth may occur in the recovery phase, with subsequent thymic enlargement, called rebound thymic hyperplasia [17]. Similar thymic enlargement has been described in patients after remission of Cushing’s syndrome, where the thymic atrophy is due to high levels of glucocorticoids in plasma, followed by rebound hyperplasia when these levels drop [16]. Following this mechanism, in dogs with neoplasia, as with the adult population described in this study, the thymus should be atrophied and therefore either barely or not visible at CT examination. Thymic hyperplasia was considered more likely than a neoplastic infiltration in our study population, but the mechanism is likely different from the one described in human medicine. We can hypothesize that at the time of presentation, these dogs were already chronically ill, and therefore, they could have passed the phase of thymic atrophy and could have already started the ’rebound’ phase. Unfortunately, given the lack of CT examination prior to the description of these findings, this hypothesis is only one of the possible explanations. 

This study has some additional limitations. First, no histological confirmation was available for the thymus for the included dogs; therefore, we could only hypothesize that the thymus visualized in our group of young dogs was normal and possibly hypertrophic in the adult dogs. The inclusion of patients with different diseases in the group of young dogs could have altered the results, with the possible inclusion of some dogs with thymic hyperplasia in this group. Other limitations included the low number of cases and the retrospective nature of the study. For these reasons, further studies are needed in order to confirm our findings and to understand the potential mechanisms of thymic hyperplasia and/or rebound in dogs, which seemed to partially differ from what has been described in human medicine. 

## 5. Conclusions

The thymus can also be identified at CT examination in adult dogs, in the case of our study, dogs affected by different types of neoplasia. Some CT characteristics of the thymus are different between young dogs (with different diseases) and adult dogs with neoplasia. The appearance of the thymic parenchyma is lobulated in adult dogs and homogeneous in juvenile dogs. The mean attenuation of the thymus is lower in adult dogs compared to young dogs, likely reflecting fat replacement, as described in human patients. 

## Figures and Tables

**Table 1:** CT features of the thymus in the dogs included in the study. Qualitative parameters are expressed as number (percentage) and quantitative parameters are reported as median (minimum–maximum).

	Adult Dogs (*n* = 11)	Juvenile Dogs (*n* = 20)
Shape	Triangular: 7 (64%) Elongated: 3 (27%) Bilobed: 1 (9%)	Triangular: 16 (80%) Flattened: 4 (20%)
Overall appearance	Lobulated: 11 (100%)	Homogeneous: 20 (100%)
Lateralization	Left-sided: 11 (100%)	Left-sided: 17 (85%) Midline: 2 (10%) Right-sided: 1 (5%)
Length	3.2 (2.5–8.6) cm	4.2 (1.9–6.6) cm
Width	1.4 (0.6–3.1) cm	1.7 (0.3–6.4) cm
Height	1.5 (1.1–2.5) cm	1.9 (1.3–4.1) cm
Contrast enhancement	Mild and homogeneous: 11 (100%)	Mild and homogeneous: 20 (100%)
Median pre-contrast attenuation	33 (5–71) HU	49 (19–60) HU
Median post-contrast attenuation	59 (27–78) HU	74 (53–92) HU

## Data Availability

Not applicable.
